# Local environment effects on charged mutations for developing aggregation-resistant monoclonal antibodies

**DOI:** 10.1038/s41598-020-78136-1

**Published:** 2020-12-03

**Authors:** Jihyeon Lee, Song-Ho Chong, Sihyun Ham

**Affiliations:** Department of Chemistry, The Research Institute of Natural Sciences, Sookmyung Women’s University, Cheongpa-ro 47-gil 100, Yongsan-Ku, Seoul, 04310 Korea

**Keywords:** Computational biophysics, Biophysical chemistry

## Abstract

Protein aggregation is a major concern in biotherapeutic applications of monoclonal antibodies. Introducing charged mutations is among the promising strategies to improve aggregation resistance. However, the impact of such mutations on solubilizing activity depends largely on the inserting location, whose mechanism is still not well understood. Here, we address this issue from a solvation viewpoint, and this is done by analyzing how the change in solvation free energy upon charged mutation is composed of individual contributions from constituent residues. To this end, we perform molecular dynamics simulations for a number of antibody mutants and carry out the residue-wise decomposition of the solvation free energy. We find that, in addition to the previously identified “global” principle emphasizing the key role played by the protein total net charge, a local net charge within $\sim$15 Å from the mutation site exerts significant effects. For example, when the net charge of an antibody is positive, the global principle states that introducing a positively charged mutation will lead to more favorable solvation. Our finding further adds that an even more optimal mutation can be done at the site around which more positively charged residues and fewer negatively charged residues are present. Such a “local” design principle accounts for the location dependence of charged mutations, and will be useful in producing aggregation-resistant antibodies.

## Introduction

Monoclonal antibodies are a growing class of biological therapeutics targeting a broad range of human disorders such as cancers and autoimmune diseases^1–3^. However, the development and application of antibodies as biomedicines have been severely limited due to their high propensities to aggregate under concentrated conditions^4,5^. Aggregation propensity is even more salient for fragments—variable heavy (V$_{\mathrm{H}}$) and light (V$_{\mathrm{L}}$) domains responsible for binding to antigens or fusions thereof—which are more preferable forms of the antibody-based regents^6,7^. (In the following, both the full-length antibody and the fragment shall be collectively referred to as antibody for brevity.) Several approaches have therefore been proposed to design aggregation-resistant antibodies^8,9^. Mutating surface residues to charged amino acids is among the promising strategies that enhances solubility and, hence, increases resistance to aggregation^10–14^. In this regard, the antibody’s net charge was recognized to be a key determinant of optimal charged mutations^15,16^: inserting positively charged residues was found to be more effective when the net charge is positive, and negatively charged residues take over the role if the net charge is negative. Since such mutations increment the magnitude of net charge, this was explained by the more strengthened electrostatic repulsion between antibody monomers, which in turn disfavors aggregation. On the other hand, the critical importance of the inserting position of charged mutations on the solubilizing activity was also recognized^15–17^, whose mechanism is still not well understood. This indicates the need to better understand the nature of charged mutations.

The impact of charged mutations on solubilizing activity can also be rationalized from a solvation viewpoint. Indeed, the water-induced attraction is often invoked to account for biomolecular self-assembly^18,19^. Such an interaction can be described by the solvation free energy characterizing the affinity for the solvent water^20^. While it is common to apply the concept of hydrophobicity to individual amino acids, one can argue the “overall protein hydrophobicity” in terms of the solvation free energy defined for a whole protein. In fact, we have recently demonstrated that the overall protein hydrophobicity is the key factor controlling protein aggregation propensity^21^. The same “global” design principle mentioned above—introducing positively (negatively) charged mutations enhances the aggregation resistance when the net charge is positive (negative)—has also been derived from the observation that the solvent water strikingly discriminates positive and negative charges depending on the protein net charge. This solvation perspective was recently adopted by Schäfer and coworkers^22^, who have successfully designed antibody mutants of improved solubility guided by the solvation thermodynamics analysis. On the other hand, it was also found that the variation in solvation free energy upon charged mutation markedly depends on the insertion location, which is outside the scope of the global principle. An additional guiding principle that takes into account the spatial neighbors of the mutation site is therefore necessary.

In this paper, we explore such a “local” design principle for charged mutations from detailed solvation thermodynamics analyses. We investigate the same antibody systems studied in Ref.^22^ since improved solution-state properties of those systems through the optimization of the solvation free energy have been experimentally confirmed. We also analyze the single-domain antibody for which the solubilizing activity of charged mutations was experimentally measured^16^. We conduct molecular dynamics simulations and carry out solvation free energy calculations for these systems. We then perform the residue-wise decomposition analysis of the solvation free energy^23–25^. This allows us to gain detailed knowledge on how the solvation free energy change is composed of individual contributions from constituent residues. We particularly focus on the contributions from the spatial neighbors around the mutation site, whose understanding will provide us with new insights on the inserting-location dependence of charged mutations. Thereby, we would like to derive a local design principle that will be useful in producing aggregation-resistant antibodies.

## Methods

### System description

**Figure 1:**
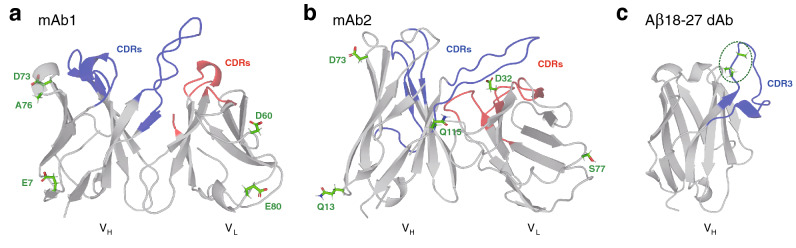
(**a**, **b**) Structures of the Fv fragments comprising variable heavy (V$_{\mathrm{H}}$) and light (V$_{\mathrm{L}}$) domains of (**a**) the wild-type mAb1 (PDB entry 3RU8) and (**b**) the wild-type mAb2 (modeled based on PDB entry 1DFB). The mutation sites studied in the present work are indicated by stick representations and green labels. Heavy and light complementarity-determining regions (CDRs) are colored blue and red, respectively. (**c**) Structure of the single-domain ($V_{\mathrm{H}}$) antibody A$\beta$18-27 dAb (modeled based on PDB entry 3B9V). In the “wild type” dAb, three Ala residues (stick representations colored green) are substituted to the mutation sites (enclosed by a green dashed oval) located at the N-terminus of the third CDR (CDR3). PyMOL version 1.8.2 (https://pymol.org) was used to generate protein figures.

We investigated the Fv fragments consisting of V$_{\mathrm{H}}$ and V$_{\mathrm{L}}$ domains of two monoclonal antibodies (mAbs), to be referred to as mAb1 and mAb2 (Fig. 1a,b). The former (mAb1) is the antibody that neutralizes human immunodeficiency virus-type 1 (HIV-1)^26^, and the latter (mAb2) is a human anti-DNA autoantibody^27^. The starting structure of mAb1 was taken from the X-ray study (PDB entry 3RU8)^28^. For mAb2, we built a homology model since no experimental structure is available. This was done with the antibody modeler module in the Molecular Operating Environment (MOE) software^29^ using the structure of PDB entry 1DFB^30^ as a template. We analyzed 10 mutants of mAb1 and 8 mutants of mAb2 introduced in Ref.^22^ (see Tables 1 and 2). The mutation sites were chosen from the solvent-exposed residues by excluding conserved residues and those within or near the complementarity-determining regions (CDRs; see Fig. 1a,b).

**Table 1:** Solvation free energy changes upon mutating mAb1.

	$\Delta Q$^a^	$\Delta G_{\mathrm{solv}}$ (kcal/mol)^b^	$\Delta G_{\mathrm{solv}}$ from Ref.^22^
**Heavy-chain mutations**			
E10G	$+\,1$	$-\,128.2$ ± 0.9	$-\,135.6$ ± 20.5
D73N	$+\,1$	$-\,85.1$ ± 16.7	$-\,87.4$ ± 19.3
A76K	$+\,1$	$-\,121.8$ ± 14.2	$-\,116.1$ ± 21.1
E10G/D73N/A76K	$+\,3$	$-\,343.8$ ± 8.4	$-\,396.9$ ± 20.1
**Light-chain mutations**			
D60S	$+\,1$	$-\,73.9$ ± 15.3	$-\,102.7$ ± 21.5
E80Q	$+\,1$	$-\,110.4$ ± 4.6	$-\,106.9$ ± 22.5
D60S/E80Q	$+\,2$	$-\,160.9$ ± 9.8	$-\,223.7$ ± 21.6
**Heavy- and light-chain mutations**			
E10G/A76K/E80Q	$+\,3$	$-\,268.0$ ± 13.3	$-\,285.7$ ± 21.5
D73N/A76K/E80Q	$+\,3$	$-\,282.4$ ± 9.8	$-\,292.3$ ± 22.0
E10G/D73N/A76K/D60S/E80Q	$+\,5$	$-\,503.1$ ± 3.5	$-\,484.0$ ± 21.1

^a^Increment in charge upon mutation(s); ^b^$\Delta G_{\mathrm{solv}} = G_{\mathrm{solv}}(\text{mutant}) - G_{\mathrm{solv}}(\text{wild } \text{type})$.

**Table 2:** Solvation free energy changes upon mutating mAb2.

	$\Delta Q$^a^	$\Delta G_{\mathrm{solv}}$ [kcal/mol]^b^	$\Delta G_{\mathrm{solv}}$ from Ref.^22^
**Heavy-chain mutations**			
Q13K	$+\,1$	$+\,12.0$ ± 13.3	$-\,1.5$ ± 13.5
D73N	$+\,1$	$-\,37.0$ ± 7.0	$-\,82.2$ ± 13.1
Q115K	$+\,1$	$-\,43.6$ ± 12.0	$-\,40.2$ ± 13.1
Q13K/D73N/Q115K	$+\,3$	$-\,218.1$ ± 11.4	$-\,216.9$ ± 14.1
**Light-chain mutations**			
D32Y	$+\,1$	$+\,60.2$ ± 19.5	$+\,56.3$ ± 12.5
S77R	$+\,1$	$+\,22.8$ ± 11.9	$-\,31.6$ ± 13.8
D32Y/S77R	$+\,2$	$-\,12.2$ ± 7.1	$+\,10.1$ ± 13.3
**Heavy- and light-chain mutations**			
Q13K/D73N/Q115K/D32Y/S77R	$+5$	$-\,316.1$ ± 20.7	$-\,205.6$ ± 13.7

^a^Increment in charge upon mutation(s); ^b^$\Delta G_{\mathrm{solv}} = G_{\mathrm{solv}}(\text{mutant}) - G_{\mathrm{solv}}(\text{wild } \text{type})$.

**Table 3:** Solvation free energy changes upon mutating A$\beta$18-27 dAb.

	$\Delta Q$^a^	$\Delta G_{\mathrm{solv}}$ [kcal/mol]^b^	Aggregation^c^
RRR-A$\beta$18-27	$+\,3$	$-\,8.5$ ± 4.5	Present
DDD-A$\beta$18-27	$-\,3$	$-\,507.7$ ± 4.0	Eliminated

^a^Increment in charge upon mutation(s); ^b^$\Delta G_{\mathrm{solv}} = G_{\mathrm{solv}}(\text{mutant}) - G_{\mathrm{solv}}(\text{wild } \text{type})$; ^c^Presence/absence of the aggregation as reported in Ref.^16^.

We also studied the single-domain (V$_{\mathrm{H}}$) antibody (dAb), to be referred to as A$\beta$18-27 dAb, which incorporates hydrophobic residues 18-VFFAEDVGSN-27 taken from the Alzheimer’s amyloid-$\beta$ (A$\beta$) peptide within the third CDR (CDR3; see Fig. 1c). A$\beta$18-27 dAb binds to A$\beta$ oligomers and fibrils with submicromolar affinity^31^, but it is also prone to self-aggregate within days at 25 $^{\circ }$C^16^. We analyzed 2 mutants of A$\beta$18-27 dAb in which three charged residues are inserted at the N-terminus of CDR3, whose solubilizing activity was measured experimentally (Table 3)^16^. The starting structure of A$\beta$18-27 dAb was also modeled with the MOE software^29^ using the structure of PDB entry 3B9V^32^ as a template.

### Molecular dynamics simulations

We used the pmemd.cuda module in AMBER18 package^33^ to perform explicit-water molecular dynamics simulations for all the systems. The ff14SB force field^34^ and TIP3P model^35^ were employed for proteins and water, respectively. The simulations were done under neutral pH, where Glu and Asp carry a negative charge and Lys and Arg a positive charge. The resulting net charges of mAb1, mAb2 and A$\beta$18-27 dAb are + 6, + 3 and 0, respectively. The whole charge of each simulation system was neutralized by counter Cl$^{-}$ ions, and additional Na$^{+}$ and Cl$^{-}$ were included to achieve a 150 mM ionic concentration. We applied the particle mesh Ewald method^36^ to handle long-range Coulomb interactions, and short-range interactions were treated by a 10 Å cutoff. Berendsen’s thermostat and barostat^37^ were used for constant temperature and pressure (300 K and 1 bar). Two independent 100 ns production simulations were carried out for each system.

### Solvation free energy analysis

Based on the general expression for the solvation free energy $G_{\mathrm{solv}}$ known as the Kirkwood charing formula, we have derived the following exact atomic decomposition of $G_{\mathrm{solv}}$^23^:

1 $$\begin{aligned} G_{\mathrm{solv}} = \sum _{\alpha } G_{\mathrm{solv}, \, \alpha } \end{aligned}$$

with

2 $$\begin{aligned} G_{\mathrm{solv}, \, \alpha } = 4 \pi \sum _{\gamma } \int _{0}^{1} d\lambda \, \int r^{2} dr \, \frac{\partial u_{\alpha \gamma }(r; \lambda )}{\partial \lambda } \, \rho _{\gamma } g_{\alpha \gamma }(r; \lambda ) \end{aligned}$$

Here $\alpha$ and $\gamma$ refer to the solute (protein) and solvent sites, respectively; $\lambda$ is the coupling parameter that introduces the solute-solvent interaction $u_{\alpha \gamma }(r)$ such that $u_{\alpha \gamma }(r; \lambda = 0) = 0$ and $u_{\alpha \gamma }(r; \lambda = 1) = u_{\alpha \gamma }(r)$; $\rho _{\gamma }$ is the average number density of site $\gamma$; and $g_{\alpha \gamma }(r; \lambda )$ is the solute-solvent radial distribution function corresponding to $u_{\alpha \gamma }(r; \lambda )$. (In the original work^23^, $\lambda$ consists of two parameters, $\lambda = (\lambda _{1}, \lambda _{2})$, to separately control the short-range and Coulomb interactions, but such a complexity is suppressed here for simplicity.) By an appropriate grouping of contributions from constituent atoms ($G_{\mathrm{solv}, \, \alpha }$), one obtains a residue-wise decomposition of $G_{\mathrm{solv}}$. In the present work, the protein-solvent distribution function was computed from the three-dimensional reference interaction site model (3D-RISM) theory^38,39^ (see the Supplementary Information for details).

## Results

### Structural and solvation free energy changes upon mutations in mAb1 and mAb2

We performed explicit-water molecular dynamics simulations for the wild-type and 10 mutants of mAb1 (Table 1) and for the wile-type and 8 mutants of mAb2 (Table 2). Protein structures were stable during the simulation time (100 ns) in all the systems: the C$_{\alpha }$ RMSD (root-mean-square deviation) from the respective initial structure stayed within $\sim$3 Å. We then computed the solvation free energy $G_{\mathrm{solv}}$ for each system using the simulated structures sampled with a 1 ns interval. In the following, we will focus on the change in solvation free energy upon mutation defined by $\Delta G_{\mathrm{solv}} = G_{\mathrm{solv}}(\mathrm{mutant}) - G_{\mathrm{solv}}(\mathrm{wild \,\, type})$. The average and standard error of $\Delta G_{\mathrm{solv}}$ were estimated based on the two independent production runs. The results for mAb1 and mAb2 are presented in Tables 1 and 2, along with the comparison with the previous work. (Results of individual trajectories are reported in Supplementary Table S1 and S2.) Overall, our numerical results for $\Delta G_{\mathrm{solv}}$ are very similar to those reported previously^22^, though some differences are discernible. In particular, $\Delta G_{\mathrm{solv}}$ values for the Q13K, S77R and D32Y/S77R mutants of mAb2 show the opposite signs compared to the previous work (Table 2). To examine such numerical differences and how they affect the main points (in particular, a local design principle) of the present work, we have carried out two additional independent 100 ns simulations for these systems, and the results are summarized in Supplementary Tables S3 and S4. As demonstrated there, the results from the additional simulations are in better agreement with the previous work, especially concerning the signs of $\Delta G_{\mathrm{solv}}$ values. This indicates that, with two independent 100 ns simulations, there still remains non-negligible uncertainty in numerical results for $\Delta G_{\mathrm{solv}}$. We will come back to this issue later in Discussion section, where we argue that our main points are nevertheless not significantly altered.

### Single-point mutations in mAb1

**Figure 2:**
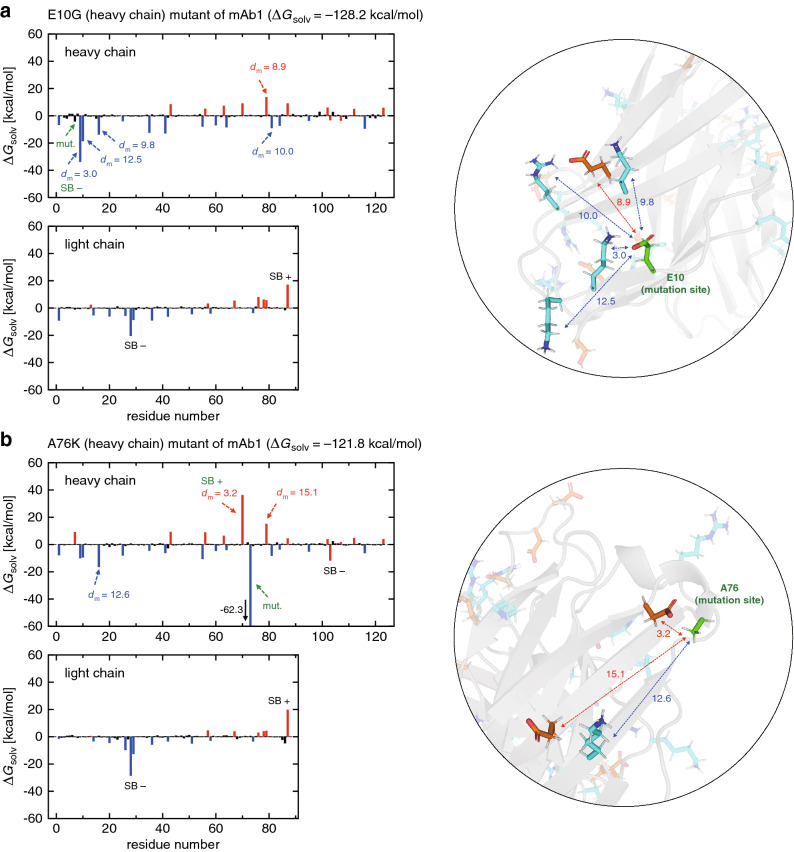
(**a**, **b**) Residue-wise decomposition of $\Delta G_{\mathrm{solv}}$ for (**a**) E10G and (**b**) A76K mutants of mAb1 (blue, red and black colors refer to positively charged, negatively charged and neutral residues, respectively). The mutation site is indicated by the green arrow. $d_{\mathrm{m}}$ (in Å) denotes the distance to the mutation site as illustrated in the inset (green stick representation, mutation site; cyan and orange representations, positively and negatively charged residues, respectively). Residues involved in the formation/breaking of salt-bridges upon mutation are represented by SB $+/-$ (green if the mutation site is involved, and black otherwise). PyMOL version 1.8.2 (https://pymol.org) was used to generate protein figures.

We start from analyzing the single-point mutants of mAb1. Individual residue contributions to $\Delta G_{\mathrm{solv}}$ upon the E10G (heavy chain) mutation are shown in Fig. 2a; those upon the A76K (heavy chain) mutation in Fig. 2b; and the results for the D73N (heavy chain), D60S (light chain) and E80G (light chain) mutations are displayed in Supplementary Fig. S1. The single-point mutations considered here are either the mutation of a negatively charged residue to a neutral one or the mutation of a neutral residue to a positively charged one, which increments the system charge by +1. (We recall here that the total charge of the wild-type mAb1 is + 6.) Hence, according to the aforementioned global principle^21^, positively charged residues get more favorably solvated (resulting in more negative $\Delta G_{\mathrm{solv}}$), whereas negatively charged residues become less favorably solvated (more positive $\Delta G_{\mathrm{solv}}$). This explains why the contributions to $\Delta G_{\mathrm{solv}}$ from the positively charged residues (colored blue) tend to exhibit negative changes and those from the negatively charged residues (colored red) show the opposite trend.

On the other hand, the presence of charged residues that exhibit more pronounced variations is discernible (indicated by the dashed arrows or SB $+/-$ in Fig. 2 and Supplementary Fig. S1). We first recognize that such large variations tend to occur in the same chain into which the mutation is introduced: for example, in the heavy-chain E10G mutation (Fig. 2a), most of the large changes show up in the heavy chain, but not in the light chain. This indicates that the distance to the mutation site matters. We therefore added the distance information ($d_{\mathrm{m}}$ in Å; the minimum side-chain heavy atom distance to the mutation site) in Fig. 2 and Supplementary Fig. S1. In fact, we find that the residues exhibiting the large solvation free energy charges are located within $\sim$15 Å from the mutation site. (In this regard, we notice that those distances, and the insets in Fig. 2, were obtained using the wile-type mAb1 structure. This is because we are interested in recipes for designing aggregation-resistant mutants that can be utilized solely based on the knowledge of the wild-type protein.) Second, we observe a sizable difference in the solvation free energy change at the mutation site (indicated by the green arrow) between the E10G (Fig. 2a) and A76K (Fig. 2b) mutants. This implies that the contents of the mutation (such as a deletion of negatively charged residue versus an addition of positively charged residue) influence $\Delta G_{\mathrm{solv}}$ value at the mutation site. Finally, it is seen that the residues involved in the formation/breaking of salt-bridges (indicated by SB $+/-$, respectively) upon mutation are significantly affected. However, except for those directly associated with the mutation site (SB $+/-$ colored green), it is difficult to predict in advance the formation/breaking of salt-bridges far from the mutation site (SB $+/-$ colored black). Therefore, those salt-bridges will not be taken into consideration in our exploration of design principles.

The key to understanding the pronounced variations in $\Delta G_{\mathrm{solv}}$ near and at the mutation site lies in the solvation structure^21^. Since the net charge of mAb1 is positive (+ 6), the protein-water electrostatic interaction induces such a long-distance orientational distribution of water where the dipole moment is directed outward from the protein. Those water molecules in turn produce an electrostatic potential that energetically favors positive charges and disfavors negative ones on the protein surface. When a charged mutation of +1 is introduced, such an electrostatic potential strengthens around the mutation site. This explains the large variations in $\Delta G_{\mathrm{solv}}$, including their signs, of the residues located within $\sim$15 Å from the mutation site. When this mutation is a deletion of a negatively charged residue, there is no charged residue that is affected by the strengthened electrostatic potential at the mutation site. On the other hand, if the mutation is an addition of a positively charged residue, the mutation site fully appreciates the strengthened electrostatic potential. This accounts for the large difference in $\Delta G_{\mathrm{solv}}$ values at the mutation site between the E10G and A76K mutants. The results for the other mutants shown in Supplementary Fig. S1 can be rationalized in a similar manner.

### Single-point mutations in mAb2

We next investigate the single-point mutants of mAb2. In contrast to large negative $\Delta G_{\mathrm{solv}}$ values ($-\,73.9$ to $-\,128.2$ kcal/mol; see Table 1) exhibited by the single-point mutants of mAb1, corresponding $\Delta G_{\mathrm{solv}}$ values of mAb2 are much less negative (the most negative value is $-\,43.6$ kcal/mol) and even take positive values (Table 2). The less negative $\Delta G_{\mathrm{solv}}$ for mAb2 can be understood from the smaller net charge of the wild-type mAb2 (+3) than that of mAb1 (+6). Indeed, according to the simple continuum Born model^40^, the solvation free energy change caused by increasing a charge from *Q* to $Q + \Delta Q$ ($\Delta Q > 0$) is proportional to $- (2Q \Delta Q + \Delta Q^{2})$, and it is negative when $Q > 0$ and it becomes more negative for larger *Q*. Thus, the positive solvation free energy change upon increasing the net charge observed for the mutants of mAb2 is counter-intuitive.

**Figure 3:**
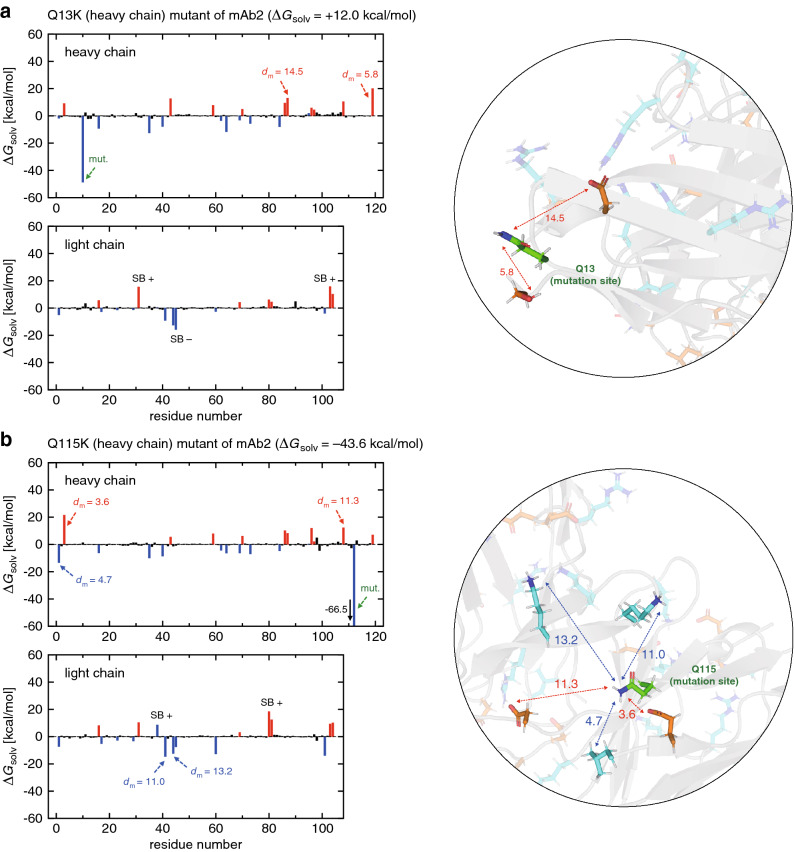
(**a**, **b**) Residue-wise decomposition of $\Delta G_{\mathrm{solv}}$ for (**a**) Q13K and (**b**) Q115K mutants of mAb2 (blue, red and black colors refer to positively charged, negatively charged and neutral residues, respectively). The mutation site is indicated by the green arrow. $d_{\mathrm{m}}$ (in Å) denotes the distance to the mutation site as illustrated in the inset (green stick representation, mutation site; cyan and orange representations, positively and negatively charged residues, respectively). Residues involved in the formation/breaking of salt-bridges upon mutation are represented by SB $+/-$ (green if the mutation site is involved, and black otherwise). PyMOL version 1.8.2 (https://pymol.org) was used to generate protein figures.

To understand such a nontrivial solvation behavior of mAb2, we analyze in detail the Q13K (heavy chain) and Q115K (heavy chain) mutants. These are the same type of mutation (glutamine to lysine), but exhibit strikingly contrasting $\Delta G_{\mathrm{solv}}$ values ($+\,12.0$ and $-\,43.6$ kcal/mol, respectively) depending on the mutation location, and, hence, constitute good examples for elucidating the position dependence of charged mutations. Residue-decomposed $\Delta G_{\mathrm{solv}}$ values for these mutants are shown in Fig. 3a,b. Main features of Fig. 3a,b can be rationalized in the same way as we explained above for mAb1: since mAb2’s net charge is positive, positive (negative) residues tend to exhibit negative (positive) solvation free energy changes; more pronounced effects show up in those residues located within $\sim$15 Å from the mutation site; and the $\Delta G_{\mathrm{solv}}$ value at the mutation site is large negative since a new positively charged residue is introduced there. However, an inspection of Fig. 3a for Q13K and Fig. 3b for Q115K reveals a difference in the distribution of charged residues around ($<\sim 15$ Å) the respective mutation site: the Q13K mutation site is surrounded by two negatively charged residues, whereas the Q115K mutation site by two negatively charged residues and three positively charged residues. Those nearby negatively charged residues provide significant positive contributions to $\Delta G_{\mathrm{solv}}$, and this accounts for the positive $\Delta G_{\mathrm{solv}}$ of the Q13K mutant. On the other hand, in the case of Q115K, such large positive contributions to $\Delta G_{\mathrm{solv}}$ arising from the nearby negatively charged residues are sufficiently compensated by large negative contributions from the nearby positively charged residues. Thus, a local net charge within $\sim$15 Å from the mutation site exerts significant impacts on the $\Delta G_{\mathrm{solv}}$ value. This also provides an explanation of the location dependence of charged mutation from the solvation perspective.

Individual residue contributions to $\Delta G_{\mathrm{solv}}$ of the other single-point mutants of mAb2—D73N (heavy chain; $\Delta G_{\mathrm{solv}} = -37.0$ kcal/mol), D32Y (light chain; $\Delta G_{\mathrm{solv}} = +60.2$ kcal/mol), and S77R (light chain; $\Delta G_{\mathrm{solv}} = +22.8$ kcal/mol) shown in Supplementary Fig. S2—can be rationalized in a similar manner. $\Delta G_{\mathrm{solv}}$ for the D73N mutant is largely negative since the mutation site is surrounded by three positively charged residues. $\Delta G_{\mathrm{solv}}$ for the D32Y mutant is positive mainly because a bulky side chain is introduced at the solvent exposed residue, which produces a large positive $\Delta G_{\mathrm{solv}}$ at the mutation site. This suggests that such a mutation that introduces a solvent-exposed bulky side chain should be avoided. $\Delta G_{\mathrm{solv}}$ for the S77R mutant is positive since the mutation site is surrounded by two positively charged residues and three negatively charged residues.

### Multipoint mutations in mAb1 and mAb2

**Figure 4:**
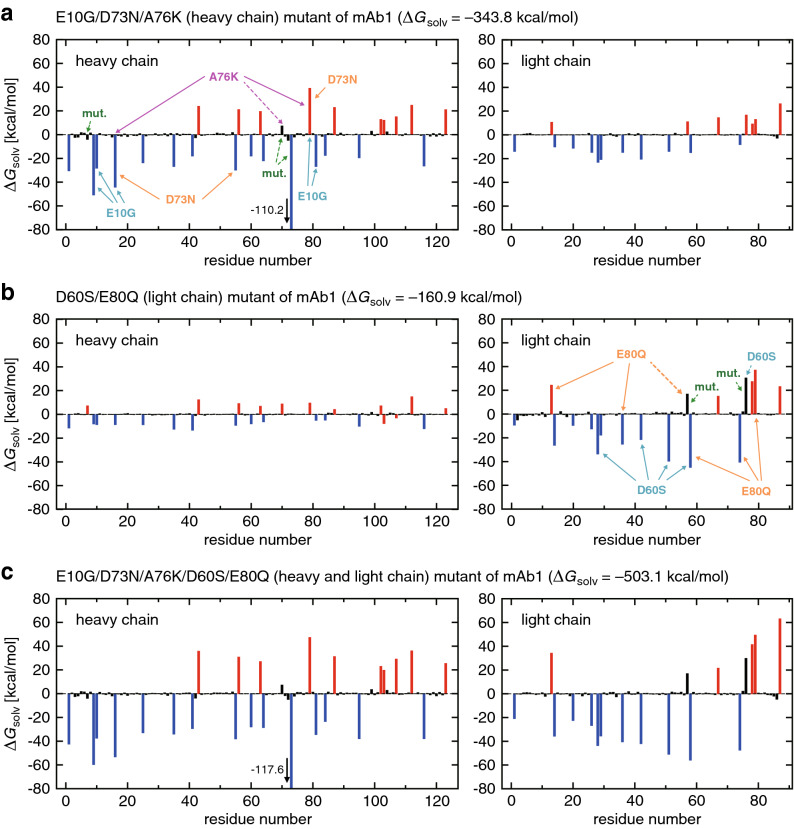
(**a**–**c**) Residue-wise decomposition of $\Delta G_{\mathrm{solv}}$ for (**a**) E10G/D73N/A76K, (**b**) D60S/E80Q, and (**c**) E10G/D73N/A76K/D60S/E80Q mutants of mAb1 (blue, red and black colors refer to positively charged, negatively charged and neutral residues, respectively). The mutation sites are indicated by the green arrows. Charged residues that exhibit pronounced variations in individual single-point mutations (taken from Fig. 2 and Supplementary Fig. S1) are represented by the solid arrows labeled with respective mutations. When those charged residues are deleted upon multipoint mutations, the solid arrows are replaced by the dashed ones.

Finally, we deal with the multipoint mutations. Because of the larger ($\ge +2$) increment of the net charge in these mutations, the protein-water electrostatic effects on $\Delta G_{\mathrm{solv}}$ become stronger. Therefore, positive (negative) residues exhibit more pronounced negative (positive) solvation free energy changes than those observed in the single-point mutations. This is seen, e.g., in Fig. 4a showing individual residue contributions to $\Delta G_{\mathrm{solv}}$ of the E10G/D73N/A76K (heavy chain) mutant of mAb1. We also notice from this figure that the solvation free energy changes induced by the three mutations can roughly be described as a superposition of the effects caused by individual mutations: the five residues strongly affected by the single E10G mutation (indicated by the dashed arrows in Fig. 2a) are shown here with the cyan arrows; the three residues associated with the A76K mutation (taken from Fig. 2b) are represented by the magenta arrows; and the three residues whose variations are large in the D73N mutant (Supplementary Fig. S1) are indicated by the orange arrows. (A certain complexity arises here since a residue that exhibits a large solvation free energy change upon one mutation may be deleted in another mutation. Such a case is exemplified by the dashed magenta arrow in Fig. 4a.) The corresponding result for the D60S/E80Q mutations in the light chain of mAb1 (Fig. 4b) can be rationalized likewise in terms of the individual D60S and E80Q mutations shown in Supplementary Fig. S1. The result for the E10G/D73N/A76K/D60S/E80Q mutations (Fig. 4c) can also be roughly described as a superposition of those shown in Fig. 4a,b, and this applies to the other (E10G/A76K/E80Q and D73N/A76K/E80Q) multipoint mutants of mAb1 (Supplementary Fig. S3).

**Figure 5:**
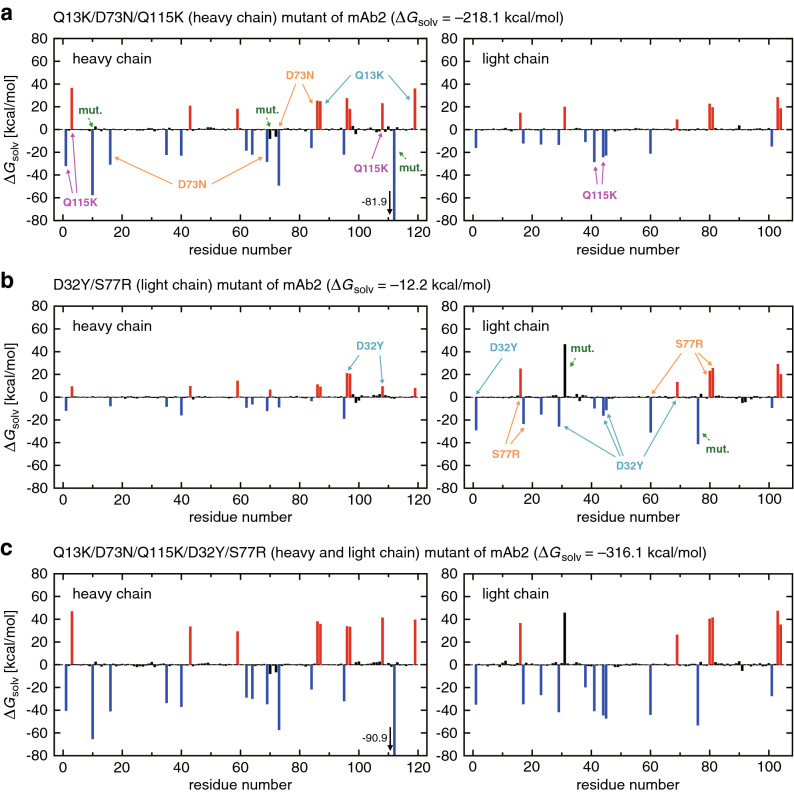
(**a**–**c**) Residue-wise decomposition of $\Delta G_{\mathrm{solv}}$ for (**a**) Q13K/D73N/Q115K, (**b**) D32Y/S77R, and (**c**) Q13K/D73N/Q115K/D32Y/S77R mutants of mAb2 (blue, red and black colors refer to positively charged, negatively charged and neutral residues, respectively). The mutation sites are indicated by the green arrows. Charged residues that exhibit pronounced variations in individual single-point mutations (taken from Fig. 3 and Supplementary Fig. S2) are represented by the solid arrows labeled with respective mutations.

The residue-wise decomposition of $\Delta G_{\mathrm{solv}}$ for the multipoint mutants of mAb2, shown in Fig. 5, can be understood similarly. The solvation free energy change for the D32Y/S77R mutant ($\Delta G_{\mathrm{solv}} = -12.2$ kcal/mol) is still not large negative in spite of the double-point charged mutation. This is not only because the bulky side chain (from D32Y) is involved, but also because five positively charged residues and six negatively charged residues are present close to those mutation sites (Fig. 5b). Upon introducing additional charged mutations, more positively charged residues from the Q13K/D73N/Q115K mutations (Fig. 5a) come into play, and the solvation free energy change for the Q13K/D73N/Q115K/D32Y/S77R mutant becomes a large negative value (Fig. 5c).

### Multipoint mutations in A$\beta$18-27 dAb

Now let us turn our attention to A$\beta$18-27 dAb. We studied the RRR-A$\beta$18-27 and DDD-A$\beta$18-27 mutants in which three charged residues (RRR or DDD) are inserted at the N-terminus of CDR3 (Fig. 1c). For the computation of $\Delta G_{\mathrm{solv}}$ and its residue-wise decomposition, we also analyzed AAA-A$\beta$18-27 (i.e., three alanine residues are substituted to the mutation sites) which was considered as the wild-type. We carried out two independent 100 ns molecular dynamics simulations for these systems, and the simulation structures were stable for all the systems (C$\alpha$-RMSD stayed within $\sim$3 Å). We then computed the solvation free energy change $\Delta G_{\mathrm{solv}} = G_{\mathrm{solv}}(\mathrm{mutant}) - G_{\mathrm{solv}}(\mathrm{wild \,\, type})$, and the results are summarized in Table 3 (see also Supplementary Table S5).

**Figure 6:**
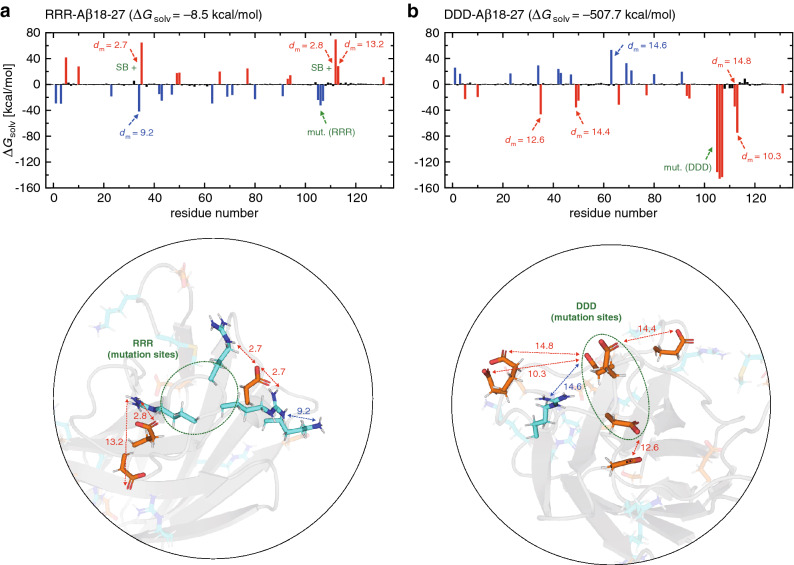
(**a**, **b**) Residue-wise decomposition of $\Delta G_{\mathrm{solv}}$ for (**a**) RRR-A$\beta$18-27 and (**b**) DDD-A$\beta$18-27 mutants of A$\beta$18-27 dAb (blue, red and black colors refer to positively charged, negatively charged and neutral residues, respectively). The mutation sites are indicated by the green arrow. $d_{\mathrm{m}}$ (in Å) denotes the distance to the mutation sites as illustrated in the inset (green dashed oval, mutation sites; cyan and orange stick representations, positively and negatively charged residues, respectively). Residues involved in the formation/breaking of salt-bridges upon mutation are represented by SB $+/-$ (green if the mutation sites are involved, and black otherwise). PyMOL version 1.8.2 (https://pymol.org) was used to generate protein figures.

The net charge of the wild-type A$\beta$18-27 dAb is 0, and its change is symmetrical for RRR-A$\beta$18-27 ($+\,3$) and DDD-A$\beta$18-27 ($-\,3$). Therefore, the global principle^21^ cannot tell us which of the charged mutations leads to more favorable solvation. (This is the primary reason why this particular dAb was chosen among several related dAbs studied in Refs.^15,16^.) Interestingly, the solubilizing activity of RRR and DDD mutations was found to be contrastive; experimental measurements indicate that aggregation is still observable for RRR-A$\beta$18-27, but it is eliminated for DDD-A$\beta$18-27^16^. Correspondingly, we find that $\Delta G_{\mathrm{solv}}$ for DDD-A$\beta$18-27 is strikingly more negative than the one for RRR-A$\beta$18-27 (Table 3). Thus, there must be some local environment effects that distinguish positively and negatively charged residues at the N-terminus of CDR3. The residue-wise decomposition analysis of $\Delta G_{\mathrm{solv}}$ is suited to address such local effects, and the results for RRR-A$\beta$18-27 and DDD-A$\beta$18-27 are shown in Fig. 6. We first note that, since the total charge of RRR-A$\beta$18-27 is positive, positive and negative residues respectively tend to exhibit negative and positive $\Delta G_{\mathrm{solv}}$ values (Fig. 6a), whereas this trend is inverted for DDD-A$\beta$18-27 (Fig. 6b). We observe from Fig. 6a,b and the insets that more negatively charged residues are present than positively charged ones within $\sim$15 Å from the mutation sites. This explains why the $\Delta G_{\mathrm{solv}}$ values at the mutation sites in RRR-A$\beta$18-27 is much less negative than those in DDD-A$\beta$18-27. Thus, it is the local principle, derived above from the analyses of mAb1 and mAb2, that accounts for the distinct solubilizing activity of RRR and DDD mutations.

## Discussion

Protein aggregation has been an area of major focus in its own right because of its relation to human diseases^41^. Factors promoting aggregation-prone nature have therefore been intensively investigated, and several algorithms have come out to rationalize and predict protein aggregation propensity^42–45^. These algorithms mainly focus on sequence characteristics such as the $\beta$-sheet forming propensity and the amino acid hydrophobicity. They have also been applied to identify aggregation-prone regions in antibodies, and it was found that such regions are typically located within CDRs responsible for antigen binding^46–48^. On the other hand, we have recently proposed a different type of strategy from a solvation viewpoint^21^. This is natural since whether a protein becomes aggregation-prone or remains soluble in aqueous environments should critically depend on the affinity for water. In fact, it was demonstrated that the solvation free energy is the key factor controlling the protein aggregation propensity^21^. Since the solvation free energy is largely affected by charged residues, we could thereby derive a design principle for increasing the resistance to aggregation through charged mutations.

However, the previously derived principle is a global one in the sense that it takes into consideration only the global net charge of a protein and the sign (positive or negative) of charged mutations. It therefore cannot discriminate, e.g., the Q13K and Q115K mutants of mAb2 studied in the present work, which however exhibit strikingly contrasting solvation free energy changes ($+\,12.0$ and $-\,43.6$ kcal/mol, respectively; see Table 2). Through the residue-wise decomposition analysis of the solvation free energy changes, it is elucidated that the contrasting behavior of the Q13K and Q115K mutants is caused by the difference in local charge surrounding the mutation site. Thus, the spatial distribution of nearby ($<\sim$ 15 Å) charged residues is found to significantly impact the solvation free energy change. We also observe that the effects of multipoint mutations can be roughly described as a superposition of those from single-point mutations. Furthermore, our analysis for mAb2 reveals why multiple mutations (more than two) are sometimes necessary to confer sufficiently negative solvation free energy. These results are in accord with the experimental observation that multiple mutations are necessary to considerably improve aggregation resistance and their effects are largely additive^17^.

Antibodies of neutral net charge are also the systems for which the previously derived global principle alone is insufficient: it cannot propose which type (positive or negative) of charged mutations should be introduced to enhance the solubility. In this connection, it was observed experimentally that inserting negatively charged residues (DDD) is more beneficial than inserting positively charged residues (RRR) in preventing the aggregation of A$\beta$18-27 dAb of neutral net charge^16^. This observation cannot be rationalized by the electrostatic repulsion since its magnitude is the same between positive charges and between negative charges. On the other hand, we demonstrate that the solvation free energy clearly discriminates DDD-A$\beta$18-27 and RRR-A$\beta$18-27 and that it can be accounted for by the local environment surrounding the mutation sites. This indicates a potential advantage of adopting the solvation viewpoint in arguing protein aggregation.

Finally, let us make comments related to the convergence of solvation free energy calculations. As stated at the beginning of Results section, additional two independent 100 ns simulations were carried out for the Q13K, S77R and D32Y/S77R mutants of mAb2, and non-negligible differences were found between the average $\Delta G_{\mathrm{solv}}$ values computed from the original and additional simulations (Supplementary Table S3). To examine the discrepancies, we plotted $\Delta G_{\mathrm{solv}}$ as a function of simulation time for individual trajectories (Supplementary Figs. S4, S5 and S6). We observe that $\Delta G_{\mathrm{solv}}$ values fluctuate significantly (the standard deviation in individual trajectories is $\sim$70 to 90 kcal/mol). Such large fluctuations in individual trajectories reflect the fact that the solvation free energy depends on fine details of the underlying protein structure: for example, the formation of just a single salt-bridge in a protein can lead to $\sim$50 kcal/mol variation^23^. Therefore, even subtle differences in the population of hydrogen bonds and salt-bridges in different trajectories can significantly affect the average $\Delta G_{\mathrm{solv}}$ value, and it is extremely difficult to obtain its converged result (e.g., within an error of $\sim$1 kcal/mol). The average $\Delta G_{\mathrm{solv}}$ values reported in the present work were computed from relatively short (100 ns) simulations, with which typical errors are of the order of $\sim$10 kcal/mol (Tables 1, 2 and 3). Hence, the average $\Delta G_{\mathrm{solv}}$ values for the Q13K, S77R and D32Y/S77R mutants of mAb2 are not decisive from our calculations: $\Delta G_{\mathrm{solv}} \sim \pm 10$ kcal/mol for these mutants with errors of the order of $\sim$10 kcal/mol.

Nevertheless, this observation does not invalidate the present work. First, the global and local effects on $\Delta G_{\mathrm{solv}}$ upon charged mutation—the main points of our work—are not affected. This is also demonstrated in Supplementary Figs. S4, S5 and S6, where the residue-wise decompositions of $\Delta G_{\mathrm{solv}}$ computed from the original and additional simulations are compared. We observe that the global effect is conserved: positive (negative) residues tend to exhibit negative (positive) solvation free energy changes in both the the original and additional simulations, and this can be explained by the fact that the net charge of the mutants under consideration is positive. In addition, the same local effects are observed in both the the original and additional simulations: the charged residues within $\sim$15 Å from the mutation site(s) are affected in the same manner. Second, our goal is to provide design principles of aggregation-resistant antibodies upon charged mutations. As stated above, it is already recognized that multiple charged mutations are necessary to improve the aggregation resistance^17^. For the multiple charged mutations, the typical magnitude of $\Delta G_{\mathrm{solv}}$ significantly exceeds 100 kcal/mol (see Tables 1, 2 and 3), which is reliable even with errors of $\sim 10$ kcal/mol. Thus, although there is a problem if our work is concerned with the precise prediction of $\Delta G_{\mathrm{solv}}$, the convergence of the global and local effects is attained already with the simulations reported here and our computational methods are reliably applicable in designing aggregation-resistant antibodies. (Mutants for which $\Delta G_{\mathrm{solv}} \sim \pm 10$ kcal/mol, such as Q13K, S77R and D32Y/S77R mentioned above, can simply be excluded from candidates of aggregation-resistant antibodies).

## Conclusions

In summary, we explore here strategies for designing aggregation-resistant antibodies by charged mutations. This is done on the basis of a solvation thermodynamics perspective rather than focusing on the sequence characteristics. By combining our previous work and the analyses presented here, we identify three critical factors that significantly impact charged mutations: (1) the total net charge of an antibody; (2) the contents of mutation (the choice of the positively or negatively charged residues and avoiding the use of residues of bulky side chains); and (3) the local net charge within $\sim$15 Å from the mutation site. The third factor accounts for the location dependence of charged mutations, and needs to be taken into consideration in searching for an optimal insertion location. A prominent advantage of charged mutations is that they can be inserted outside the complementarity-determining regions mediating target binding, i.e., the antibody solubility can in principle be improved without reducing the binding affinity. We hope that our design principles will be useful in producing aggregation-resistant antibody therapeutics.

## Supplementary information

Supplementary Information
