# Supplementary material for: Local environment effects on charged mutations for developing aggregation-resistant monoclonal antibodies
Source: Sci Rep. 2020 Dec 3;10:21191. doi: 10.1038/s41598-020-78136-1 (PMC7713239; doi:10.1038/s41598-020-78136-1)
Supplement: Supplementary file 1 — Supplementary Information [file 41598_2020_78136_MOESM1_ESM.pdf]

Supplementary Information for

Local Environment Effects on Charged Mutations for Developing Aggregation-Resistant Monoclonal Antibodies

Jihyeon Lee, Song-Ho Chong, and Sihyun Ham*

Department of Chemistry, The Research Institute of Natural Sciences,
Sookmyung Women's University,
Cheongpa-ro 47-gil 100, Yongsan-ku, Seoul 04310, Korea

Corresponding author:

*Sihyun Ham

**Address**	Department of Chemistry
	The Research Institute of Natural Sciences
	Sookmyung Women's University
	Cheongpa-ro 47-gil 100, Yongsan-ku, Seoul 04310, Korea
**Email**	sihyun@sookmyung.ac.kr
**Phone**	+82-2-710-9410
**Fax**	+82-2-2077-7321

Methods

Protein-solvent distribution function

For a given protein conformation taken from simulations and a specified protein-solvent interaction potential (its λ dependence shall be suppressed here for notational simplicity), we can compute the protein-solvent distribution function using the three-dimensional reference interaction site model (3D-RISM) theory.[S1,S2] In this theory, the 3D distribution function $g_\gamma(\mathbf{r})$ of the solvent site γ at position $\mathbf{r}$ around a protein is obtained by self-consistently solving the 3D-RISM equation

$$h_\gamma(\mathbf{r}) = \sum_{\gamma'} \int d\mathbf{r}' \, \chi_{\gamma\gamma'}(|\mathbf{r} - \mathbf{r}'|) \, c_{\gamma'}(\mathbf{r}') \tag{S1}$$

and the closure relation

$$h_\gamma(\mathbf{r}) = \begin{cases} \exp[d_\gamma(\mathbf{r})] - 1 & \text{for} \quad d_\gamma(\mathbf{r}) \le 0 \\ d_\gamma(\mathbf{r}) & \text{for} \quad d_\gamma(\mathbf{r}) > 0 \end{cases} \tag{S2}$$

where $d_\gamma(\mathbf{r}) = -u_\gamma(\mathbf{r})/(k_\mathrm{B}T) + h_\gamma(\mathbf{r}) - c_\gamma(\mathbf{r})$. Here $h_\gamma(\mathbf{r}) = g_\gamma(\mathbf{r}) - 1$ refers to the 3D total correlation function of the solvent site γ; $c_\gamma(\mathbf{r})$ is the corresponding direct correlation function; $\chi_{\gamma\gamma'}(r)$ denotes the site-site solvent susceptibility function, treated as an input to the theory; and $u_\gamma(\mathbf{r})$ is the interaction potential generated by protein atoms. The same numerical procedure as described in Ref. S2 was employed to solve the above equations along with the susceptibility function for the solvent model calculated from the dielectrically consistent RISM theory.[S3] The radial distribution function $g_{\alpha\gamma}(r)$ between the protein atom α and the solvent site γ can then be obtained by shifting the origin of the coordinate to the position $\mathbf{r}_\alpha$ of the atom α followed by the orientational average, i.e.,

$$g_{\alpha\gamma}(r) = \frac{1}{4\pi} \int d\hat{\mathbf{r}} \, g_\gamma(\mathbf{r}_\alpha + \mathbf{r}) \tag{S3}$$

with $\hat{\mathbf{r}} = \mathbf{r}/r$ and $r = |\,\mathbf{r}\,|$.

References

(S1) Kovalenko, A. Three-Dimensional RISM Theory for Molecular Liquids and Solid-Solid Interfaces. Molecular Theory of Solvation. Dordrecht, 2003; pp 169–275.

(S2) Imai, T.; Harano, Y.; Kinoshita, M.; Kovalenko, A.; Hirata, F. A Theoretical Analysis on Hydration Thermodynamics of Proteins. *J. Chem. Phys.* **2006**, *125*, 024911.

(S3) Perkyns, J.; Pettitt, B. M. A Site-Site Theory for Finite Concentration Saline Solutions. *J. Chem. Phys.* **1992**, *97*, 7656–7666.

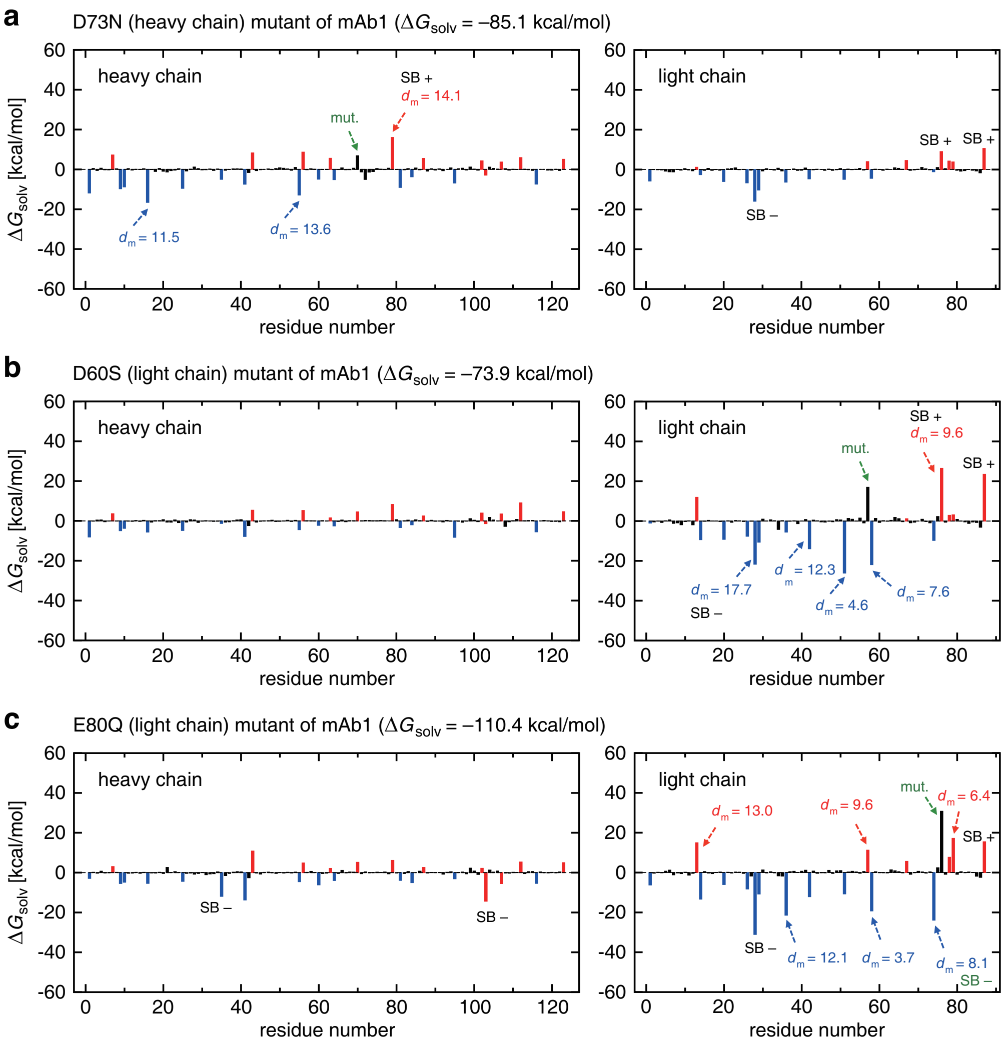

Figure S1: (a–c) Residue-wise decomposition of ΔG_{solv} for (a) D73N, (b) D60S, and (c) E80Q mutants of mAb1 (blue, red and black colors refer to positively charged, negatively charged and neutral residues, respectively). The mutation site is indicated by the green arrow. d_{m} (in Å) denotes the distance to the mutation site. Residues involved in the formation/breaking of salt-bridges upon mutation are represented by SB +/− (green if the mutation site is involved, and black otherwise).

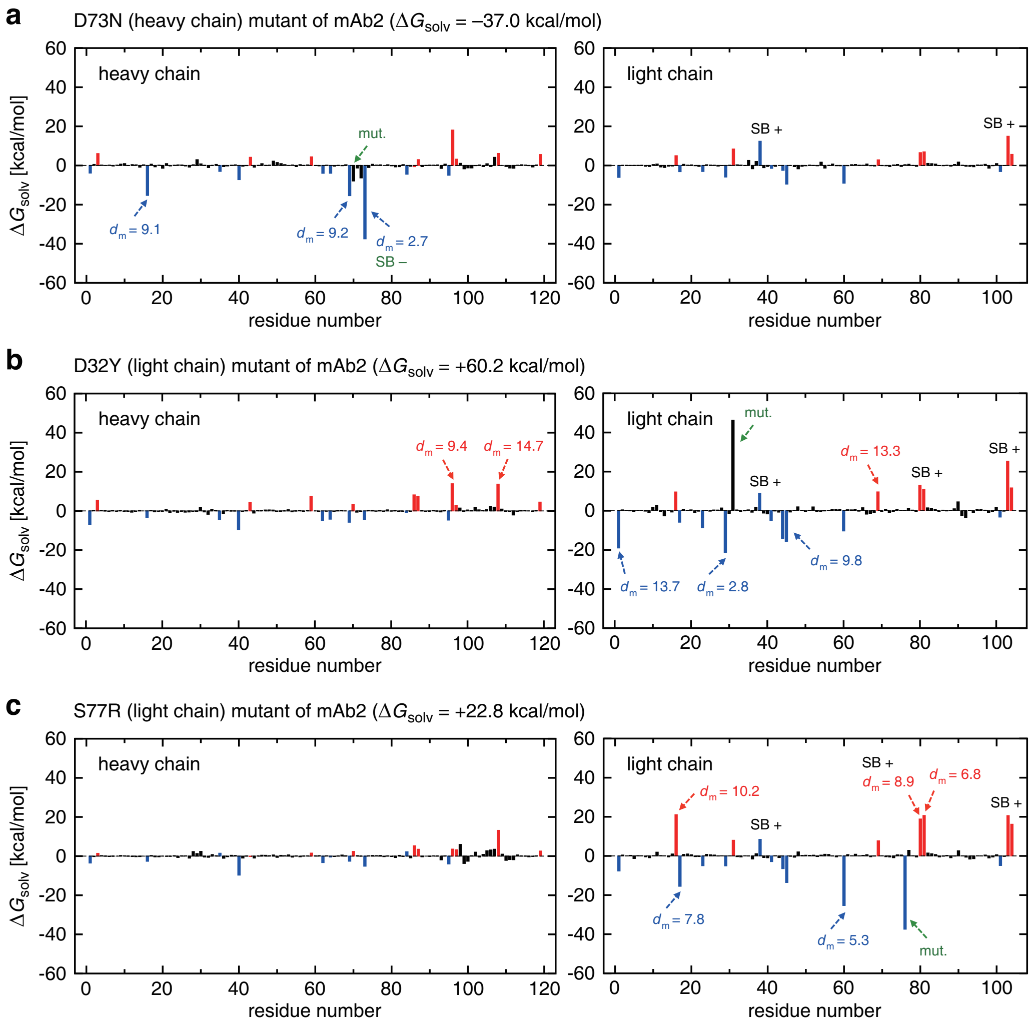

Figure S2: (a–c) Residue-wise decomposition of ΔG_{solv} for (a) D73N, (b) D32Y, and (c) S77R mutants of mAb2 (blue, red and black colors refer to positively charged, negatively charged and neutral residues, respectively). The mutation site is indicated by the green arrow. d_{m} (in Å) denotes the distance to the mutation site. Residues involved in the formation/breaking of salt-bridges upon mutation are represented by SB +/− (green if the mutation site is involved, and black otherwise).

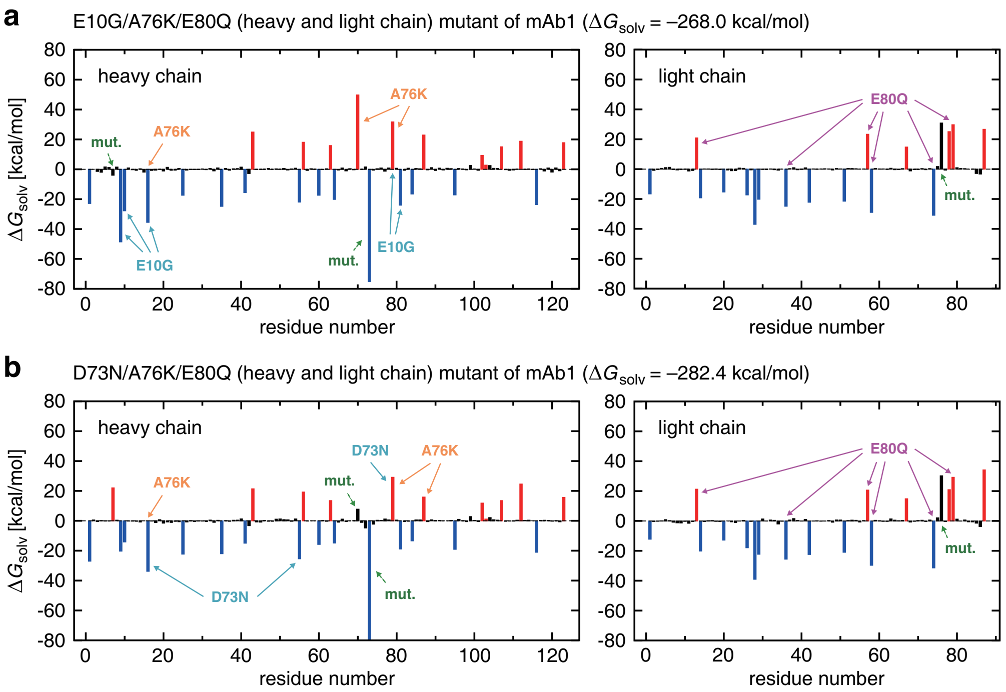

Figure S3: (a,b) Residue-wise decomposition of ΔG_{solv} for (a) E10G/A76K/E80Q and (b) D73N/A76K/E80Q mutants of mAb1 (blue, red and black colors refer to positively charged, negatively charged and neutral residues, respectively). The mutation sites are indicated by the green arrows. Charged residues that exhibit pronounced variations in individual single-point mutations (taken from Fig. 2 and Fig. S1) are represented by the solid arrows labeled with respective mutations.

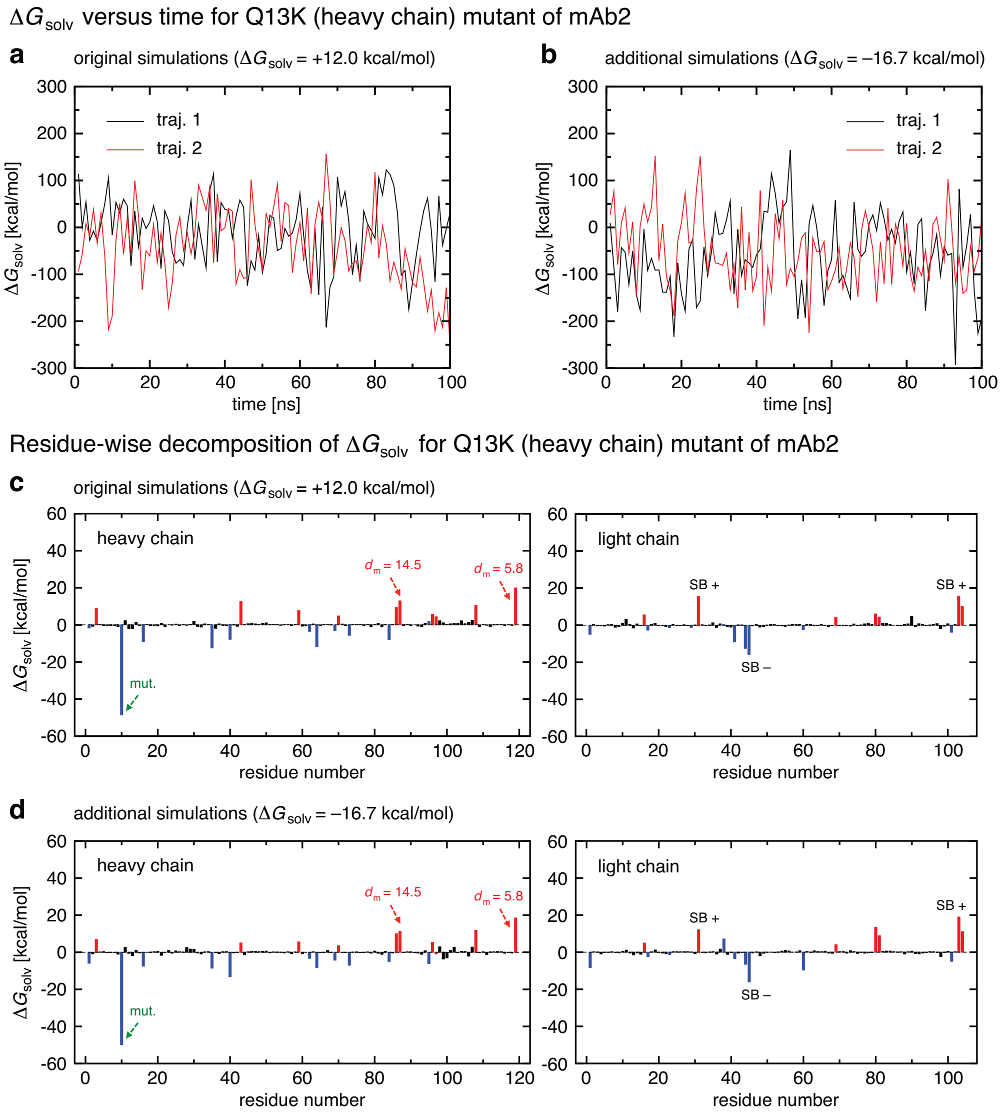

Figure S4: Original versus additional simulations for the Q13K mutant of mAb2. (a,b) ΔG_{solv} values for protein configurations along (a) the original simulation trajectories and (b) the additional simulation trajectories (b). (c,d) Residue-wise decomposition of ΔG_{solv} (blue, red and black colors refer to positively charged, negatively charged and neutral residues, respectively) based on (c) the the original simulation trajectories (identical to Fig. 3a) and (d) the additional simulation trajectories. The mutation site is indicated by the green arrow. d_{m} (in Å) denotes the distance to the mutation site. Residues involved in the formation/breaking of salt-bridges upon mutation are represented by SB +/− (green if the mutation site is involved, and black otherwise).

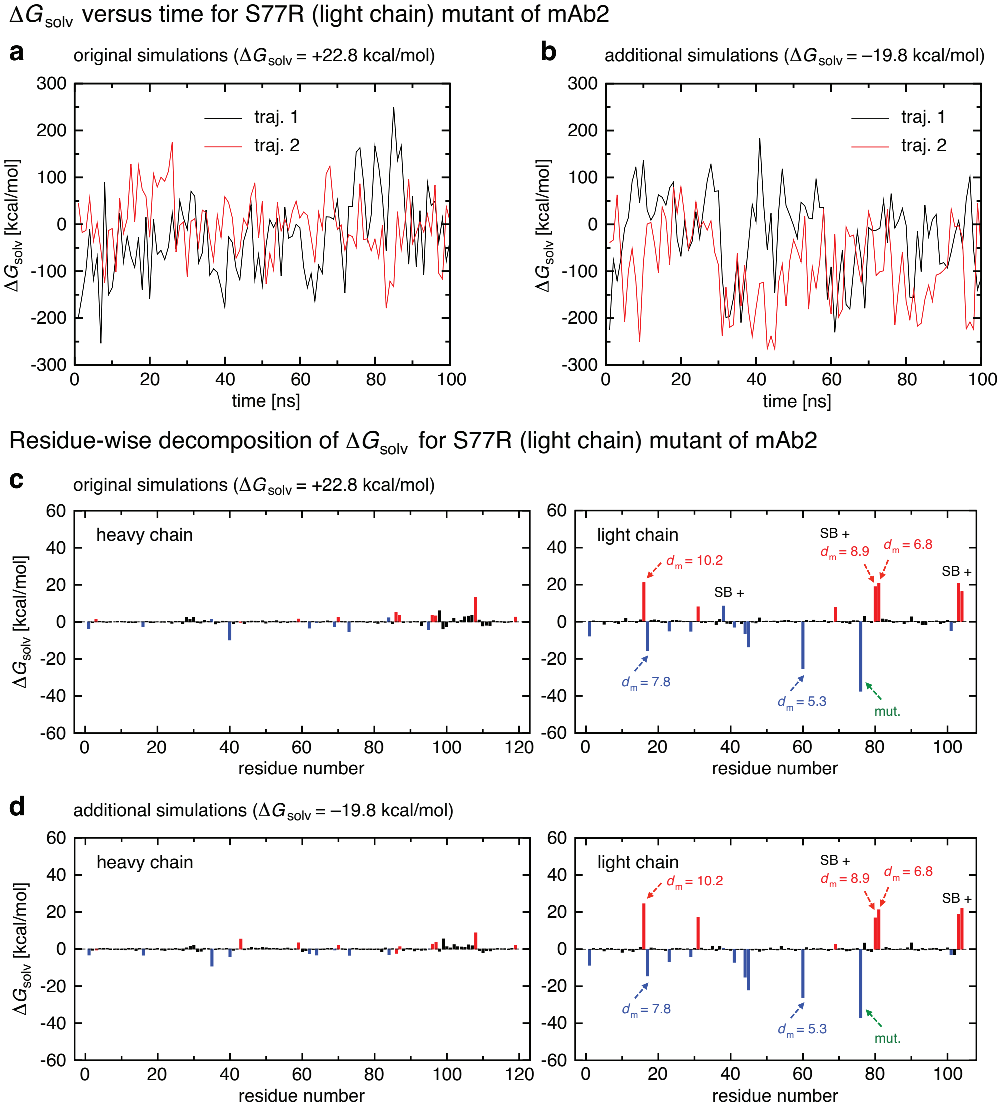

Figure S5: Original versus additional simulations for the S77R mutant of mAb2. (a,b) ΔG_{solv} values for protein configurations along (a) the original simulation trajectories and (b) the additional simulation trajectories (b). (c,d) Residue-wise decomposition of ΔG_{solv} (blue, red and black colors refer to positively charged, negatively charged and neutral residues, respectively) based on (c) the the original simulation trajectories (identical to Fig. S2c) and (d) the additional simulation trajectories. The mutation site is indicated by the green arrow. d_{m} (in Å) denotes the distance to the mutation site. Residues involved in the formation/breaking of salt-bridges upon mutation are represented by SB +/− (green if the mutation site is involved, and black otherwise).

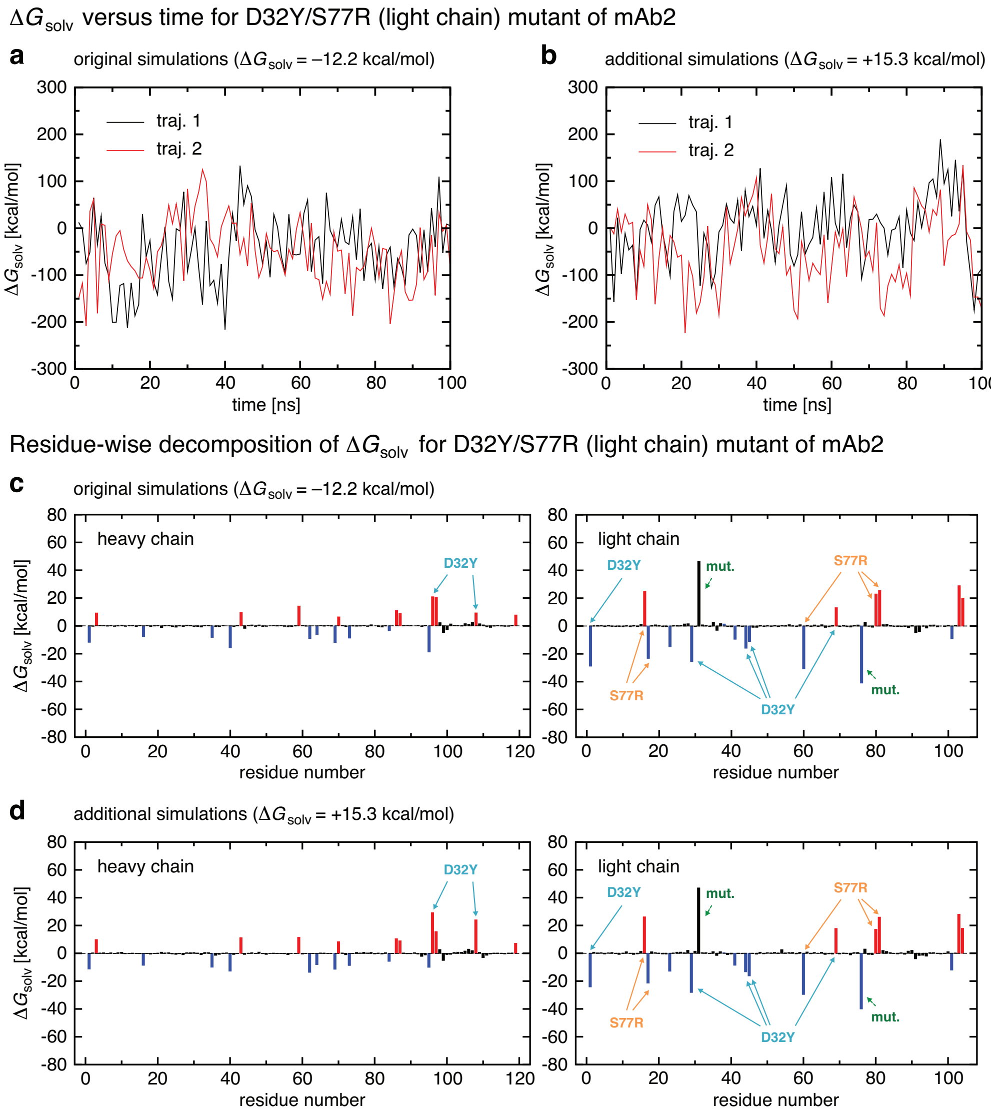

Figure S6: Original versus additional simulations for the D32Y/S77R mutant of mAb2. (a,b) ΔG_{solv} values for protein configurations along (a) the original simulation trajectories and (b) the additional simulation trajectories (b). (c,d) Residue-wise decomposition of ΔG_{solv} (blue, red and black colors refer to positively charged, negatively charged and neutral residues, respectively) based on (c) the the original simulation trajectories (identical to Fig. 5b) and (d) the additional simulation trajectories. The mutation site is indicated by the green arrow. d_{m} (in Å) denotes the distance to the mutation site. Residues involved in the formation/breaking of salt-bridges upon mutation are represented by SB +/− (green if the mutation site is involved, and black otherwise).

Table S1: Solvation free energy changes upon mutating mAb1 in individual trajectories

	trajectory 1	trajectory 2	ΔG_{solv} [kcal/mol] [a]
heavy-chain mutations			
E10G	−128.9	−127.6	−128.2 ± 0.9
D73N	−108.7	−61.4	−85.1 ± 16.7
A76K	−101.7	−141.8	−121.8 ± 14.2
E10G/D73N/A76K	−331.9	−355.7	−343.8 ± 8.4
light-chain mutations			
D60S	−95.5	−52.3	−73.9 ± 15.3
E80Q	−104.0	−116.9	−110.4 ± 4.6
D60S/E80Q	−174.7	−147.0	−160.9 ± 9.8
heavy- and light-chain mutations			
E10G/A76K/E80Q	−249.2	−286.8	−268.0 ± 13.3
D73N/A76K/E80Q	−268.6	−296.1	−282.4 ± 9.8
E10G/D73N/A76K/D60S/E80Q	−498.3	−507.9	−503.1 ± 3.5

[a] Average ± standard error.

Table S2: Solvation free energy changes upon mutating mAb2 in individual trajectories

	trajectory 1	trajectory 2	ΔG_{solv} [kcal/mol] [a]
heavy-chain mutations			
Q13K	+28.0	−4.1	+12.0 ± 13.3
D73N	−37.6	−36.3	−37.0 ± 7.0
Q115K	−29.9	−57.3	−43.6 ± 12.0
Q13K/D73N/Q115K	−205.3	−230.9	−218.1 ± 11.4
light-chain mutations			
D32Y	+85.9	+34.4	+60.2 ± 19.5
S77R	+9.2	+36.4	+22.8 ± 11.9
D32Y/S77R	−10.1	−14.3	−12.2 ± 7.1
heavy- and light-chain mutations			
Q13K/D73N/Q115K/D32Y/S77R	−288.6	−343.7	−316.1 ± 20.7

[a] Average ± standard error.

Table S3: Original versus additional simulation results for ΔG_{solv} upon mutating mAb2

	original simulations [a] ΔG_{solv} [kcal/mol] [c]	additional simulations [b] ΔG_{solv} [kcal/mol] [c]	ΔG_{solv} of Ref. 22 [d]
heavy-chain mutations			
Q13K	$+12.0 \pm 13.3$	-16.7 ± 7.7	-1.5 ± 13.5
light-chain mutations			
S77R	$+22.8 \pm 11.9$	-19.8 ± 25.0	-31.6 ± 13.8
D32Y/S77R	-12.2 ± 7.1	$+15.3 \pm 18.8$	$+10.1 \pm 13.3$

[a] Original simulation results reported in the main text; [b] Additional simulation results; [c] $\Delta G_{\text{solv}} = G_{\text{solv}}(\text{mutant}) - G_{\text{solv}}(\text{wild type})$; [d] ΔG_{solv} taken from Ref. 22 in the main text.

Table S4: Solvation free energy changes upon mutating mAb2 in individual trajectories of additional simulations

	trajectory 3	trajectory 4	ΔG_{solv} [kcal/mol] [a]
heavy-chain mutations			
Q13K	−21.5	−11.9	−16.7 ± 7.7
light-chain mutations			
S77R	+14.1	−53.7	−19.8 ± 25.0
D32Y/S77R	+40.1	−9.4	+15.3 ± 18.8

[a] Average ± standard error.

Table S5: Solvation free energy changes upon mutating Aβ18-27 dAb in individual trajectories

	trajectory 1	trajectory 2	ΔG_{solv} [kcal/mol] [a]
RRR-Aβ18-27	-5.3	-11.7	-8.5 ± 4.5
DDD-Aβ18-27	-506.6	-508.7	-507.7 ± 4.0

[a] Average $\pm$ standard error.
